# ^125^I seed brachytherapy for non-central pelvic recurrence of cervical cancer after external beam radiotherapy

**DOI:** 10.1186/s13014-024-02454-1

**Published:** 2024-06-08

**Authors:** Xuemin Di, Zhen Gao, Huimin Yu, Xiaoli Liu, Jinxin Zhao, Juan Wang, Hongtao Zhang

**Affiliations:** Department of Oncology, Hebei General Hospital, 348 West Heping Road, Shijiazhuang, Hebei 050051 China

**Keywords:** Brachytherapy, Non-central pelvic recurrence of Cervical Cancer, External Beam Radiotherapy, Locoregional, Pelvic recurrence

## Abstract

**Objective:**

To investigate the efficacy of ^125^I seed brachytherapy for non-central pelvic recurrence of cervical cancer after external beam radiotherapy, and to analyze the clinical influential factors.

**Methods:**

Between June 2015 and April 2022, 32 patients with 41 lesions were treated with ^125^I seed brachytherapy. The seeds were implanted under the guidance of CT and/or 3D-printed template images at a median dose of 100 Gy (range, 80–120 Gy), and the local control rate (LCR) and survival rates were calculated. We used multivariate logistic regression to identify prognosis predictors, and receiver operating characteristic (ROC) curve analysis to determine the optimal cut-off values.

**Results:**

The median follow-up was 48.52 months (range, 4–86 months), and the 6-, 12-, and 24-month LCR was 88.0%, 63.2%, and 42.1%, respectively. The 1- and 2-year survival rates were 36% and 33%, respectively, and the median survival time was 13.26 months. No significant adverse events occurred. Multivariate regression analysis showed that tumor diameter, tumor stage, and LCR were independent factors influencing survival. ROC curve analysis showed that the area under the curve for tumor diameter and D90 were 0.765 and 0.542, respectively, with cut-off values of 5.3 cm and 108.5 Gy.

**Conclusions:**

The present findings indicate that ^125^I seed brachytherapy is feasible for treating non-central pelvic recurrence of cervical cancer after external beam radiotherapy. Further, tumor diameter < 5.3 cm and immediate postoperative D90 > 108.5 Gy were associated with better efficacy.

## Introduction

Cervical cancer is one of the most common gynecological tumors in women [1]. The standard management of patients with early cervical cancer is surgery and/or external beam radiotherapy with or without chemotherapy. However, cervical cancer eventually relapses in most cases. The recurrence rate of stage IB–IIA cervical cancer (according to the staging system of the International Federation of Gynecology and Obstetrics) is 11–22%, while the recurrence rate of stage IIB–IVA cancer is 28–64% [2, 3]. Among patients in whom radiotherapy is not successful, pelvic recurrence occurs in 60% and relapse occurs in 80% within 2 years after treatment [2, 3]. Failure of radical treatment of cervical cancer is associated with a poor prognosis, and the 5-year survival rate in such cases is only 10–20% [4–6].

Treatment of recurrent cervical cancer is currently a challenge, especially for those with a history of radiotherapy. Resection is not possible in most cases of cervical cancer recurrence due to local expansion, post-radiation fibrosis, local and distant metastasis, and other contraindications such as the likelihood of an unacceptable level of dysfunction after resection [7]. Stereotactic body radiotherapy is an emerging technology for small lesions that are associated with good local control and a low incidence of serious adverse reactions [8, 9], but most reports about this method are from retrospective studies with small samples [10]. Further, re-irradiation with this method is associated with a high risk of toxicity to the rectum, intestine, and bladder, as well as grade 3 or 4 morbidities [11].

Re-irradiation with or without concurrent cisplatin-based chemotherapy is the first-line option for patients with recurrent cervical cancer after external beam radiotherapy [1]. According to the classification of recurrent cervical cancer, there are three main categories: Central type, pelvic type and pelvic appearance.However, in the case of pelvic recurrence, it is difficult to deliver adequate doses to recurrent lesions because there is a limitation on the radiation dose that normal tissue can be exposed to. Moreover, palliative chemotherapy may not be the optimal option for salvage treatment [11, 12]. Central pelvic recurrence after radiotherapy can be treated with surgery, but surgical interventions are associated with a high incidence of postoperative complications such as vaginal fistula [13, 14]. Furthermore, pelvic dissection for non-central recurrence has a high mortality rate, which limits its clinical application [15, 16]. Brachytherapy is one of the most promising salvage therapies for recurrent cervical cancer after external beam radiotherapy [17–19]. In particular, brachytherapy with ^125^I and ^192^Ir has been reported to be effective for the treatment of recurrent cervical cancer [20–22]. The overall dosimetric characteristics of the two isotopes are similar, but ^192^Ir is the preferred choice for treating central recurrence of cervical cancer, while ^125^I is preferred for non-central pelvic recurrence of cervical cancer after external beam radiotherapy [23, 24]. Moreover, brachytherapy with ^125^I seeds is considered an advanced form of low-dose radiotherapy, especially for the salvage treatment of recurrent cancer after external radiotherapy [20, 25]. Permanent interstitial ^125^I seed implantation offers the advantages of high precision, strong adaptability, and little damage to surrounding organs, and it is especially beneficial for patients who have received radiation before [26, 27]. Based on the previous findings discussed above, we propose a salvage technique using image-guided ^125^I seed brachytherapy to treat non-central pelvic recurrence of cervical cancer after external beam radiotherapy.

## Methods and materials

### Patient and tumor characteristics

We reviewed 32 cases with 41 non-central lesions of pelvic recurrent cervical cancer after external beam radiotherapy in which brachytherapy was performed at our institution between June 2015 and April 2022. The confirmation of recurrence was mostly based on imaging examination(CT、MRI、PETCT) and examination of tumor markers or biopsy.The patient and tumor characteristics are summarized in Table 1.Patients treated with standard chemoradiotherapy were treated with previous treatments, and patients treated with concurrent chemoradiotherapy were given doses of 45(40 to 50)Gy. Unresectable lymph nodes can be treated with a simultaneous dose or a delayed dose of 10 to 15 Gy by highly conformal radiotherapy. ^192^ Ir as part of initial treatment, A point A or high risk CTV(HR-CTV)is prescribed at a dose of (5 to 7)Gy(4 to 6) times for a total of 20 to 35 Gy. Only 2 of the 32 patients did not receive chemotherapy after brachytherapy because of abnormal liver and kidney function.Based on the protocol followed at our hospital, the indications for ^125^I seed brachytherapy were as follows: (1) non-central pelvic recurrent cervical cancer after external beam radiotherapy, (2) the presence of metastatic lesions that are determined to be inoperable or unresectable by an experienced gynecologist; (3) blood routine results showing a white blood cell count of ≥ 3 × 10^9^/L, a neutrophil absolute value of > 1.5 × 10^9^, a platelet count of ≥ 75 × 10^12^/L, a hemoglobin concentration of ≥ 90 g/L, and normal coagulation function; (4) a Karnofsky performance score (KPS) of 70 or higher; (5) expected survival of ≥ 3 months and (6) no major organ dysfunction. The exclusion criteria were (1) major organ dysfunction, such as severe heart, lung, liver, and kidney dysfunction; (2) severe coagulation disorder; (3) poor general condition or bad fluid quality with acute or chronic infections; and (4) less than 3 months since the end of the last external radiotherapy. All the included patients provided their written informed consent, and this study received the approval of the Institutional Review Board. Our multidisciplinary team discussed the decision to administer brachytherapy.

**Table 1:** Patient and tumor characteristics

Variable	Value/type/stage/size	No. of patients	Percentage
Age	< 50 years	17	52.1
	≥ 50 years	15	46.8
Pathological type	Squamous cell carcinoma	29	90.6
	Non-squamous cell carcinoma	3	9.3
Previous treatment regimens	Surgery + radiotherapy + chemotherapy	23	71.8
	Radiotherapy + chemotherapy Surgery + radiotherapy	5 4	15.6 12.5
Diagnostic staging	staging I	6	18.7
	staging II	4	12.5
	Staging III	15	46.8
	Staging IV	7	21.8
Maximum diameter of the tumor (lesion)	< 5.3 cm	23	71.8
	≥ 5.3 cm	9	28.1

surgery, radiotherapy and chemotherapy did not receive the three treatments at the same time

### Preoperative planning

One week before implantation, the patients were immobilized with a vacuum cushion in the treatment position. A position line was drawn along the CT positioning laser line on the surface of the patient’s skin around the tumor location, and three to four markers were pasted on the horizontal line. Then, enhanced CT scanning was performed with a slice thickness of 5 mm. Next, the Prowess treatment planning system (TPS) (Panther Brachy version 5.0 TPS; Prowess Inc., Concord, CA, USA) was used to create a Brachy Stereo–Seed preplan. During the pre-planning with the TPS, the gross target volume (GTV) is the location and extent of tumor confirmed by imaging examination (CT, MRI, PET-CT, etc.)and the organ at risk (OAR) were delineated, but the clinical target volume (CTV) was obtained by expanding the GTV by 5 mm in all directions. The organs at risk (OAR)are mainly intestine, bladder, ureter, etc., and the dose is converted into particle dose by BED and EQD2, referring to the dose of external radiotherapy.The delineations were carefully done following the CT images, and the volume of the CTV influenced the choice of seed activity (0.3–0.7 mCi). The needles were implanted, and the seeds were loaded according to a pre-plan for a patient. The median prescription dose was 100 Gy (range, 80–120 Gy), which was determined by the conversion of BED and EQD2 according to the specific organ at risk, the dose of radiotherapy and the time between radiotherapy. Finally, a dose–volume histogram was generated. A 3D template was printed with a sla600 type 3D printer (Unicorn 3DSL450M; Beijing Unicorn Science and Technology Ltd., Beijing, China) according to the biological surface characteristics of the seed implantation area, the X-axis and Y-axis laser lines, a registration mark, and information about the simulated needle path.

### Brachytherapy protocol

The patients were fixed in the same position as the preplanning stage with a vacuum cushion. Surgery was carried out with the patients under anesthesia induced by local infiltration or nerve block, and the CT positioning scan was used to select the puncture points and determine the angle and depth of the puncture path. Single-use needles were inserted into the target lesion under CT guidance by using a freehand implantation technique or a 3D-printed template, with a distance of 0.5–1.0 cm between seeds and a distance of 1 cm between needles. A CT scan was obtained to confirm that the template location was correct, and then a Mick applicator was used to implant seeds according to the preoperative plan.After seed implantation, CT was performed again to view the actual distribution of the ^125^I seeds in the target areas, and additional seeds were implanted if the ^125^I seeds were not adequately distributed within the target volume.Finally, the images were transferred into the TPS to verify the dose distribution.

### End-points and follow-up

The primary endpoint was local control rate (LCR) at 6 months, and the secondary endpoint was overall survival (OS). Multivariate logistic regression was used to determine the factors associated with treatment efficacy, and cut-off values were determined by receiver operating characteristic (ROC) curve analysis. Local tumor response was evaluated according to the Response Evaluation Criteria in Solid Tumors (RECIST1.1) criteria, and complications were scored according to the criteria set by the Radiation Therapy Oncology Group/European Organisation for Research and Treatment of Cancer Late Radiation Morbidity Score.

Follow-up assessments were performed at 1, 2, 4, 6, 9, and 12 months after the procedure, and after 1 year, the patients were followed up every 6 months. The follow-up clinical evaluations mainly included physical examination, CT scans, and magnetic resonance imaging (MRI).The total follow-up period was 5years.

### Statistical analysis

Statistical analysis was performed using SPSS version 22.0 (IBM Corp., Armonk, NY, USA). If a patient underwent particle implantation at two or three different sites, each site was considered separately when LCR was analyzed. The Kaplan-Meier method was used to estimate survival rates. The paired *t*-test was used to compare the parameters of the preoperative plan and the actual postoperative results. The Wilcoxon rank–sum test was used for univariate analysis, and the Cox regression model was used for multivariate analysis. The selected common independent variables were analyzed by ROC curve analysis, and the area under the curve (AUC) was calculated. *P* < 0.05 was considered to indicate statistical significance.

## Results

The median age of the 32 patients included in this study was 54 years (range, 32–70 years). The KPS ranged from 80 to 100, and the median follow-up time was 48.52 months (range, 4–86 months). No patients experienced in-field failure, and 23 patients (71.8%) had developed distant metastases and died.

### Treatment outcomes

**Fig. 1:**
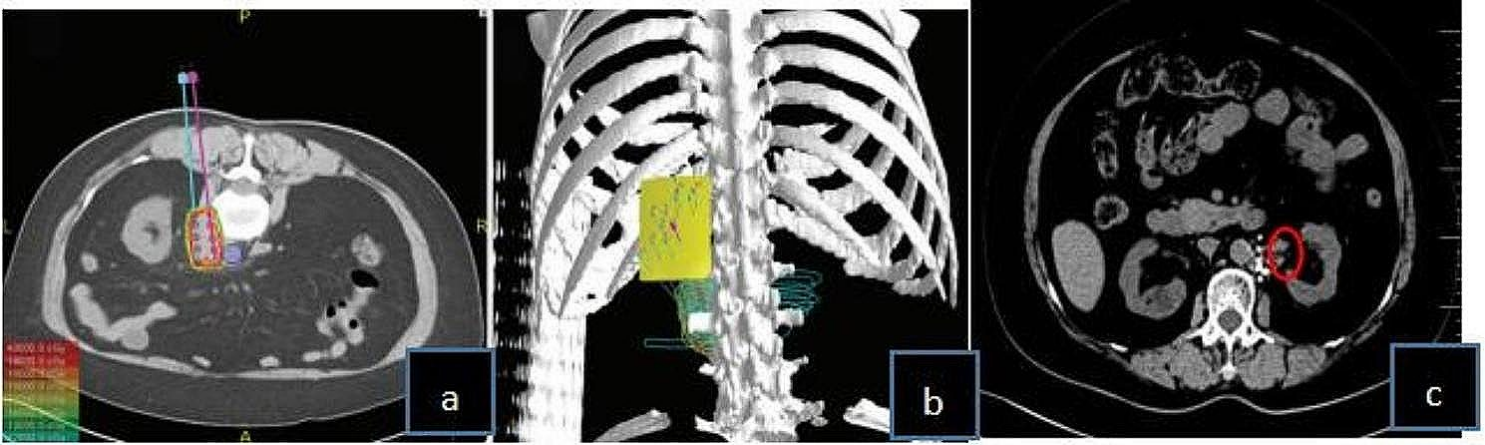
(**a**) Preplan diagram of the target area made with the treatment planning system to determine the number and location of the radioactive implanted seeds and the direction and depth of the needles. (**b**) Three-dimensional view of the reconstructed template, needles, skin surface, tumor, and organs at risk. (**c**) CT scan taken 5 years after surgery showing the tumor was locally controlled and stable

Postoperative CT re‑examination was performed after 5 years(the follow-up assessments were performed at 1, 2, 4, 6, 9, and 12 months after the procedure, and after 1 year the patients were followed up every 6 months.after 2 year the patients were followed up every 12months)and compared with the preoperative CT observations. Representative CT images taken before and after ^125^I implantation are presented in Fig. 1. The LCR for all 41 lesions was 38 out of 41 (92.6%): this included 12 lymph nodes with complete response (29.2%), 18 with partial response (43.9%), and 8 with stable disease (19.5%). Progressive disease was found in the remaining 3 lesions (0.73%).

**Fig. 2:**
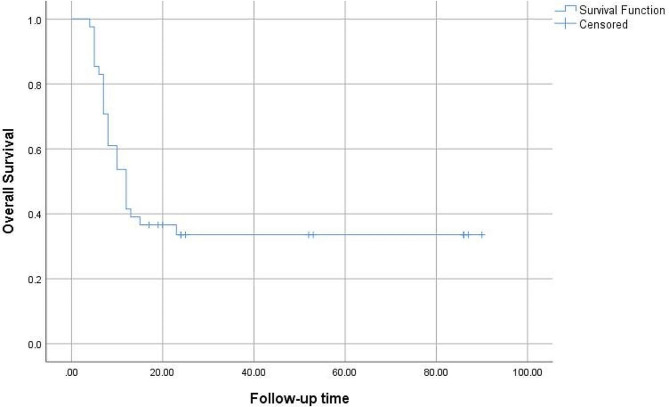
Survival curve

For the survival analysis, patients were followed up for 4–86 months. The median overall survival was 13.26 months, and the 1- and 2-year survival rates were 36% and 33%, respectively (Fig. 2).

**Fig. 3:**
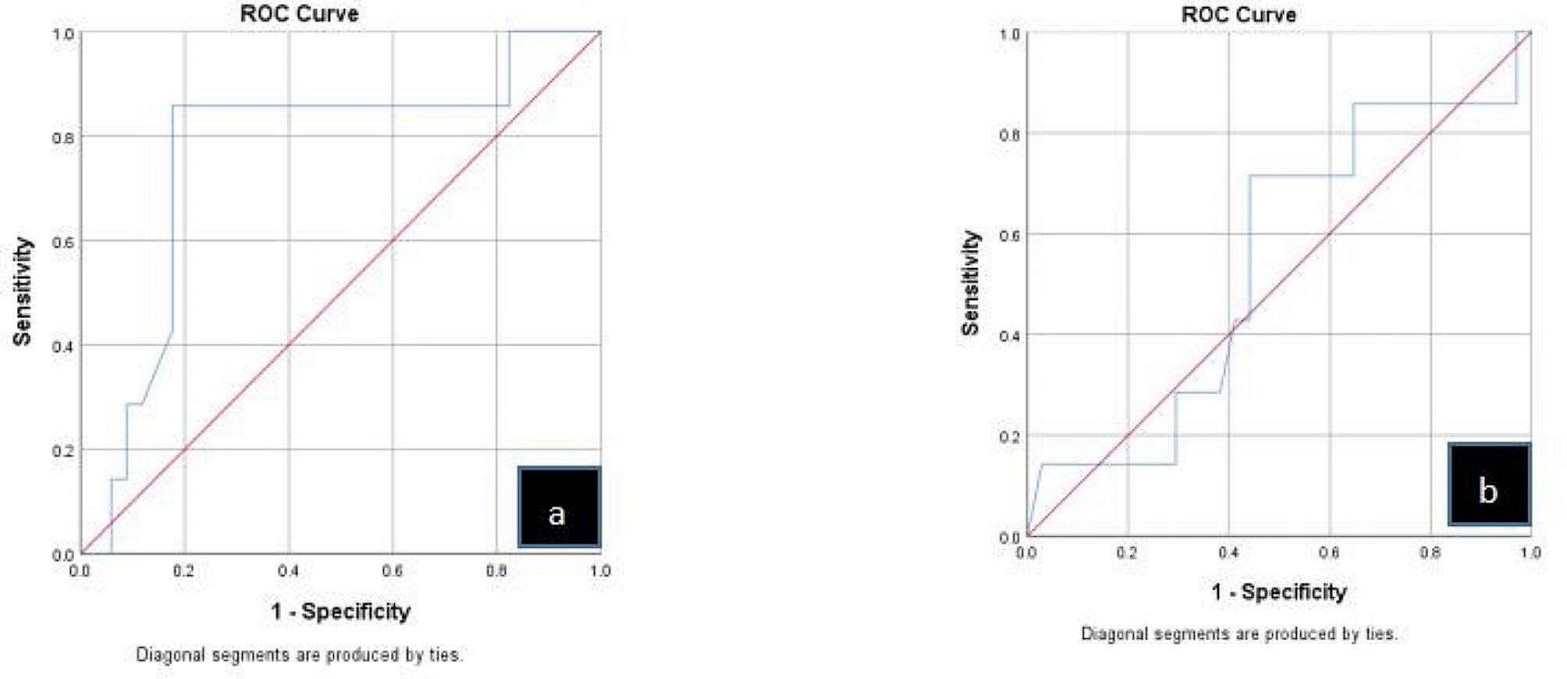
ROC curve for maximal tumor diameter and D90

### Prognostic factors

Univariate analysis showed that the longest tumor diameter was associated with local control(Table 2)These variables were used for logistic multivariate regression analysis, which showed that tumor diameter (odds ratio [OR] = 0.008, 95% confidence interval [CI], 1.018–1.683), tumor stage (OR = 0.031, 95% CI = 1.114–9.662), and LCR (OR = 0.032, 95% CI = 1.088–1.193) were independent factors that influenced survival (*P* < 0.05). (Table 3)ROC curve analysis showed that the area under the curve (AUC) for tumor diameter and D90 were 0.765 and 0.542 respectively, with cut-off values of 5.3 cm (sensitivity, 0.857; specificity, 0.824) and 108.5 Gy (sensitivity, 0.714; specificity, 0.559), respectively (Fig. 3).

**Table 2:** Univariate analysis of factors associated with local control

Factor	Number of examples(*n*)	Age (y)		Stage		Seed activity (mCi)		Longest tumor diameter (cm)	Implantation		Immediately after surgery, D90[Gy; Mean ± sd]	Immediately after surgery, D100[Gy; Mean ± sd]	Adjuvant chemotherapy (example)		Immediate postoperative V90[%]	Immediate postoperative V100[%]	Immediate postoperative V150[%]
		< 50	>=50	I-II	III-IV	< 0.5	>=0.5		unarmed	3D			Yes	No			
effective	34	18	16	11	23	18	16	4.00 ± 2.23	28	6	115.19 ± 22.50	59.63 ± 20.07	32	2	93.83 ± 4.02	89.88 ± 4.50	62.04 ± 9.31
void	7	4	3	2	5	1	6	6.21 ± 2.13	3	4	100.71 ± 24.86	64.29 ± 23.61	7	0	92.91 ± 6.57	88.74 ± 9.34	58.86 ± 15.22
The test value		< 0.001		< 0.001		2.107		-2.407	3.002		-5.111	-0.543	-		0.489	0.496	0.735
*P*-value		1.000		1.000		0.147		**0.021**	0.083		0.011	0.590	1.000		0.627	0.622	0.467

**Table 3:** Multivariate analysis of factors associated with survival

								Multivariate analyses	
Factor	Value/description	n	Median (mo)	6 mo (%)	12 mo (%)	18 mo (%)	24 mo (%)	95% CI	*P*
Age (y)	<50	22	11.25	45	41	35	35	0.329–1.664	0.466
	≥ 50	19	14.50	63	32	32	32		
Stage	I–II	13	84.00	69	62	62	62	1.114–9.662	**0.031**
	III–IV	28	11.40	82	56	48	45		
Seed activity (mCi)	<0.5	19	13.00	53	37	37	37	0.146–2.251	0.425
	≥ 0.5	22	13.44	55	36	30	30		
Implantation	unarmed	31	13.75	55	38	35	35	0.374–3.518	0.810
	3D	10	12.00	50	30	30	30		
Adjuvant chemotherapy	no	2	9.00	0	-	-	-	0.082–2.034	0.275
	yes	39	14.09	56	38	35	35		
Longest tumor diameter (cm)	<5.3	29	13.00	52	39	39	39	1.081–1.683	**0.008**
	≥ 5.3	12	13.43	56	32	25	25		
D90 (Gy)	<108.5	21	12.95	52	37	37	37	0.978–1.025	0.932
	≥ 108.5	20	13.50	55	35	30	30		
LCR	CR+PR	34	12.00	50	35	32	32	1.088–1.193	**0.032**
	SD+PD	7	16.05	71	40	40	40		

### Toxicity and complications

No major complications related to the procedure occurred during or after brachytherapy. There was a small amount of intraoperative bleeding that was successfully treated with topical hemostatic treatment. The toxicity prevalence was low in this study, so the factors that might be related to toxicity could not be evaluated.

## Discussion

In the present study, we have examined the efficacy of brachytherapy with ^125^I seeds for non-central pelvic recurrence of cervical cancer after external radiotherapy and the factors that affect the prognosis of this treatment. In our patients, the 6-, 12-, and 24-month LCR was 88.0%, 63.2%, and 42.1%, respectively, and the 1- and 2-year survival rates were 36% and 33%, respectively. Further, the median survival time was 13.26 months. The effective control rates at 1, 3, 6, and 12 months were 76.7%, 80.0%, 83.3%, and 86.7%, respectively in the 125I seed implantation group [28]. These results suggest that ^125^I brachytherapy can be successfully used to improve local control in patients with cervical cancer relapse after radiotherapy.

In the present study, multivariate regression analysis showed that tumor diameter, tumor stage, and LCR were independent factors that were significantly associated with prognosis. Similarly, Martinez-Monge et al. also found tumor diameter, tumor stage, and LCR to be significant prognostic factors in the treatment of recurrent rectal cancer with radioactive seed implantation [29]. In addition, Qu reported that colorectal adenocarcinoma recurrent in the pelvis and paraortics recurrence site, tumor volume, and radiation dose were the main factors that affected efficacy in curative effect [17].

The diameter of the tumor has always been an important factor affecting the curative effect of tumors because of its close relationship with the surrounding tissue and its influence on the vital organs, blood vessels, and ribs. It may be difficult to accurately determine the clinical tumor target area for larger tumors, as larger tumors have a higher probability of developing liquefactive necrosis and are more prone to seeds translocation. Further, it may not be possible to deliver an adequate dose to the tissue around the tumor, and this could lead to tumor recurrence. The larger the tumor diameter (or volume), the poorer is the blood supply in the tumor center, the higher is the proportion of hypoxic cells, and the greater is the resistance to radiation. Accordingly, Han reported that tumor diameter was an important factor affecting the survival rate of patients [30], as the 3-year survival rate of patients with advanced cervical cancer who had para-aortic lymph node metastases of diameter < 5 cm and > 5.0 cm was 18% and 13%, respectively. Huang [31] also believed that tumor diameter was a risk factor affecting treatment efficacy and, therefore, prognosis. In this study, ROC curve analysis indicated a cut-off value of 5.3 cm (sensitivity, 0.857; specificity, 0.824), with the one-year overall survival of patients with tumor diameter < 5.3 cm and > 5.3 cm were significantly different at 39% and 25%, respectively.

Radiation dose is a core parameter of particle implantation, which is widely used in the treatment of various recurrent solid tumors due to its advantages of dose distribution and minimal invasiveness. Yao reported that a median D90 value of 126.5 Gy (range, 100–198 Gy), a median intestinal radiation dose of 60 Gy (range, 48–66 Gy), and a median maximum radiation dose to the spinal cord of 36 Gy (range, 23.5–60 Gy) did not result in any obvious complications [32]. Qu Ang reported that D90 > 105 Gy or D100 > 91% significantly prolonged the local progression-free survival rate of pelvic recurrent cervical cancer [17]. In agreement with the previous findings, the results of the present study showed that an immediate postoperative D90 of > 108.5 Gy had a better curative effect than lower doses. This could be a reference point for the selection of prescription dose and the design of dose-escalation studies in the future.

## Conclusions

The present results indicate that ^125^I seed brachytherapy for non-central pelvic recurrent cervical cancer after external beam radiotherapy is a safe, effective, and minimally invasive option. However, these observations need to be confirmed in large-scale prospective studies in the future in order to confirm its efficacy and safety, and to determine the optimal doses and delivery strategies.

## Data Availability

The datasets used and analyzed during the present study are available from the corresponding author at the reasonable request.
